# Robot-Assisted Radical Cystectomy: Recommendations from Latin American Experts Consensus on Surgical Strategy, Urinary Reconstruction, and Oncological and Functional Outcomes

**DOI:** 10.1590/S1677-5538.IBJU.2026.0413

**Published:** 2026-07-05

**Authors:** Eliney Ferreira Faria, Alejandro Rodriguez, Alexandre Pompeo, Arnaldo Fazoli, Carlos Vaz, Carlos Watanabe, Clovis Fraga, Daher Chade, Eder Brazão, Elias Bodden, Fernando Korkes, Flávio Cárcano, Francisco Hidelbrando, Gilberto Almeida, Gustavo Guimarães, Hamilton Zampoli, Joan Palou, Jose Alexandre Pedrosa, Lucas Fornazieri, Marcelo Kerkebe, Marcio Moschovas, Marcos Tobias-Machado, Matheus Nister, Matheus Paiva, Murilo Luz, Nilo Jorge, Pablo Melo, Pedro Romanelli, Peter Wiklund, Rafael Coelho, Raphael Rocha, Rene Sotelo, Reza Mehrazin, Roberto Machado, Rodolfo Borges, Rodrigo Valente, Ronald Kool, Stenio Zequi, Sérgio Zacchi, Vipul Patel, Wenceslao Villamil, Rodrigo Gualberto

**Affiliations:** 1 Faculdade Ciências Médicas Minas Gerais Belo Horizonte MG Brasil Faculdade Ciências Médicas Minas Gerais, Belo Horizonte, MG, Brasil; 2 North Carolina Wake Forest Winston-Salem NC USA North Carolina Wake Forest, Winston-Salem, NC, USA;; 3 Hospital do Coração São Paulo SP Brasil Hospital do Coração, HCor, São Paulo, SP, Brasil;; 4 Instituto de Câncer Dr. Arnaldo Vieira de Carvalho São Paulo SP Brasil Instituto de Câncer Dr. Arnaldo Vieira de Carvalho, São Paulo, SP, Brasil; 5 Hospital Felício Rocho Belo Horizonte MG Brasil Hospital Felício Rocho, Belo Horizonte, MG, Brasil; 6 Hospital Sírio Libanês de Brasília Brasília DF Brasil Hospital Sírio Libanês de Brasília, Brasília, DF, Brasil; 7 Hospital Português Recife PE Brasil Hospital Português, Recife, PE, Brasil; 8 A. C. Camargo Cancer Center São Paulo SP Brasil A. C. Camargo Cancer Center, São Paulo, SP, Brasil; 9 Hospital Punta Pacífica Panamá Hospital Punta Pacífica, Panamá; 10 Faculdade de Medicina do ABC Santo André SP Brasil Faculdade de Medicina do ABC - FMABC, Santo André, SP, Brasil;; 11 Hospital Monte Klinikum Fortaleza CE Brasil Hospital Monte Klinikum, Fortaleza, CE, Brasil; 12 Universidade do Vale do Itajaí Itajaí SC Brasil Universidade do Vale do Itajaí - UNIVALI, Itajaí, SC, Brasil; 13 Fundació Puigvert Barcelona Spain Fundació Puigvert, Barcelona, Spain; 14 Instituto Nacional do Câncer Rio de Janeiro RJ Brasil Instituto Nacional do Câncer - INCA, Rio de Janeiro, RJ, Brasil; 15 University of Chile Santiago Chile University of Chile. Santiago, Chile; 16 Global Robotics Institute Celebration Florida USA Global Robotics Institute, Celebration, Florida, USA; 17 Hospital Madre Teresa Belo Horizonte MG Brasil Hospital Madre Teresa, Belo Horizonte, MG, Brasil; 18 Santa Casa de Santos Santos SP Brasil Santa Casa de Santos, Santos, SP, Brasil; 19 Icahn School of Medicine at Mount Sinai New York USA Icahn School of Medicine at Mount Sinai, New York, USA; 20 Rede Mater Dei Belo Horizonte MG Brasil Rede Mater Dei, Belo Horizonte, MG, Brasil, Brasil; 21 Universidade Federal de Minas Gerais Belo Horizonte MG Brasil Universidade Federal de Minas Gerais - UFMG, Belo Horizonte, MG, Brasil; 22 The Mount Sinai Hospital NY USA The Mount Sinai Hospital, NY, USA; 23 Américas Medical City Rio de Janeiro RJ Brasil Américas Medical City, Rio de Janeiro, RJ, Brasil; 24 University of Southern California Los Angeles CA USA University of Southern California - USC, Los Angeles, CA, USA; 25 Hospital de Amor Barretos SP Brasil Hospital de Amor, Barretos, SP, Brasil; 26 Faculdade de Medicina de Ribeirão Preto Ribeirão Preto SP Brasil Faculdade de Medicina de Ribeirão Preto - USP, Ribeirão Preto, SP, Brasil; 27 Erasto Gaertner Cancer Center Curitiba PR Brasil Erasto Gaertner Cancer Center, Curitiba, PR, Brasil; 28 Hospital Santa Rita de Cássia Vitória ES Brasil Hospital Santa Rita de Cássia, Vitória, ES, Brasil; 29 Hospital Italiano de Buenos Aires Argentina Hospital Italiano de Buenos Aires, CABA, Argentina

**Keywords:** Urinary Bladder Neoplasms, Cystectomy, Robotic Surgical Procedures

## Abstract

**Purpose:**

To develop contemporary evidence-informed recommendations for robot-assisted radical cystectomy (RARC), urinary reconstruction, and perioperative management, integrating current evidence with the experience of Latin American experts in uro-oncology and robotic surgery.

**Materials and methods:**

A modified Delphi consensus process was conducted involving 42 experts with extensive experience in RARC. Topics included patient selection, perioperative optimization, enhanced recovery after surgery (ERAS), lymph node dissection, urinary reconstruction, functional preservation, perioperative systemic therapy, complex clinical scenarios, and emerging robotic technologies. A comprehensive literature review was performed using Medline, Scopus, and Web of Science through November 2025, following PRISMA principles and incorporating recommendations from EAU, AUA/ASCO/SUO, and NCCN guidelines. Consensus was defined as ≥75% agreement.

**Results:**

Consensus supported routine implementation of ERAS protocols, structured frailty and nutritional assessment, extended pelvic lymph node dissection, and perioperative systemic therapy in eligible patients. Intracorporeal urinary diversion was associated with improved recovery and lower wound-related morbidity. Nerve-sparing and organ-preserving approaches were recommended in selected patients to optimize continence, sexual function, and quality of life. Orthotopic neobladder reconstruction should be individualized according to oncologic safety, functional status, renal function, and patient preference. RARC was considered feasible in complex settings, including obesity, bulky lymphadenopathy, locally advanced disease, and salvage surgery, when performed in experienced high-volume centers. Areas without consensus included urinary drainage strategies and antibiotic prophylaxis duration.

**Conclusions:**

This expert consensus provides recommendations for RARC and urinary reconstruction, aiming to standardize practice, optimize perioperative care, and improve oncological and functional outcomes. Future prospective studies are needed.

## INTRODUCTION

Radical cystectomy with urinary reconstruction remains the standard of care for patients with localized muscle-invasive bladder cancer and for selected cases of high-risk non–muscle-invasive disease refractory to conservative therapies (1, 2).

Despite these advances, robotic-assisted radical cystectomy (RARC) remains a highly complex procedure, encompassing not only extirpative surgery but also pelvic lymphadenectomy and urinary reconstruction, often performed intracorporeally. As a result, significant variability persists across institutions and surgeons regarding patient selection, surgical technique, perioperative management, and reconstructive strategies (3-5).

This heterogeneity is further compounded by differences in surgical experience, institutional volume, and the lack of standardized technical frameworks, particularly in areas such as nerve-sparing approaches, extent of lymphadenectomy, intracorporeal urinary diversion (ICUD), and management of complex cases. In addition, the learning curve associated with robot-assisted radical cystectomy represents a significant barrier to its widespread adoption, particularly outside high-volume, specialized centers. Although multiple guidelines address the oncologic management of bladder cancer, specific procedural aspects of RARC remain insufficiently standardized.

In this context, the development of an expert consensus is essential to provide structured, evidence-informed recommendations that reflect both current literature and real-world surgical practice. The aim of this consensus is to define key principles and technical considerations in RARC, identify areas of agreement and controversy, and support the standardization of care across different practice settings.

## MATERIALS AND METHODS

This consensus was developed by a multidisciplinary panel of 42 experts in urologic oncology with extensive experience in RARC. Panelists were selected based on predefined criteria, including recognized expertise in robotic surgery, high surgical volume, academic productivity, and experience in the critical appraisal of scientific literature.

A structured consensus process based on a modified Delphi methodology was conducted, consisting of a single round of online voting. Initially, key topics were identified, including patient selection, surgical technique, lymphadenectomy, urinary reconstruction, perioperative management, complications, and oncologic and functional outcomes.

A comprehensive literature review was performed using Medline, Scopus, and Web of Science databases, including studies published up to November 2025, with updates incorporated when relevant. Search terms included "robot-assisted radical cystectomy," "robotic cystectomy," and related keywords. The review followed PRISMA (Preferred Reporting Items for Systematic Reviews and Meta-Analyses) guidelines to ensure methodological rigor, transparency, and reproducibility. In addition, current international guidelines from major societies, including the NCCN, AUA, and EAU, were reviewed.

Based on the available evidence and expert input, the working groups drafted preliminary questions and consensus statements, which were subsequently distributed to the panelists for voting. Agreement was assessed using structured voting, and consensus was defined as ≥75% agreement among respondents. Statements not reaching this threshold were identified as areas of controversy and are explicitly discussed in the manuscript.

Following the voting process, the statements were refined through expert discussion, and a final set of recommendations was established, integrating both the best available evidence and panel consensus.

Finally, a designated subgroup of experts was responsible for drafting the preliminary manuscript, which was subsequently reviewed, discussed, and approved by all panel members prior to final consolidation (Figure-1).

**Figure 1:**
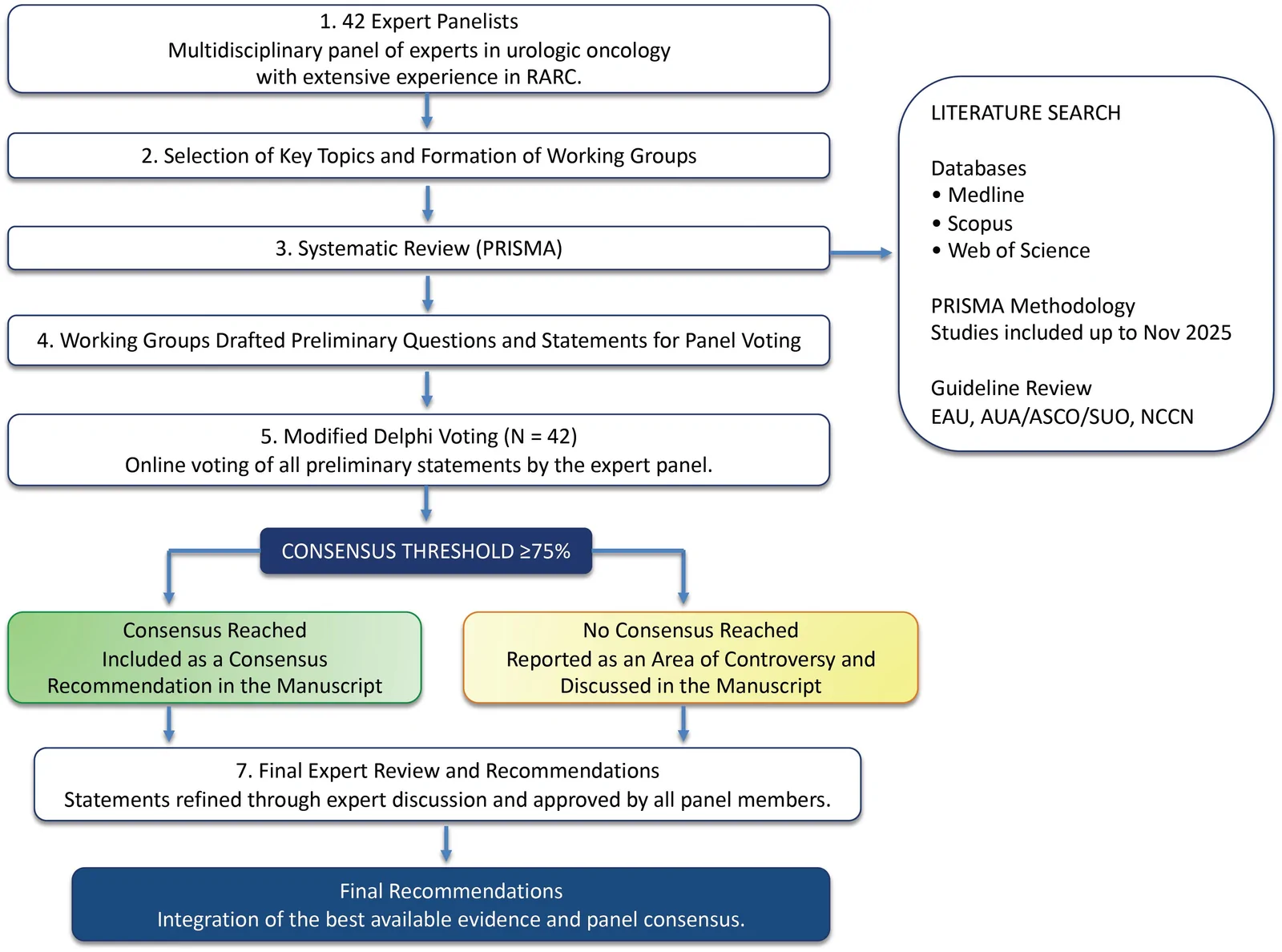
Flowchart of the modified Delphi methodology used for consensus development.

## RESULTS

### Surgical preparation

#### Patient Selection

RARC shares the same indications as the open approach, and, according to the majority of panelists, patient involvement in the decision-making process is essential when selecting the surgical approach (Table-1). There are few absolute or relative contraindications to RARC; however, certain clinical scenarios require advanced surgical expertise and should be approached with caution (6).

**Table 1:** Indications for Robot-Assisted Radical Cystectomy (RARC).

Category	Clinical Situation	Recommendation / Comment
**Primary (Curative) Indication**	Non-metastatic MIBC	RARC has the same indications as ORC for curative intent.
	Refractory or recurrent disease after BCG	Considered a valid option in cases of intravesical therapy failure.
	Extensive, high-risk, multifocal tumors or tumors not controllable with TURBT + intravesical therapy	Radical treatment with RARC.
**Muscle-invasive Variant Histologies**	Pure bladder adenocarcinoma	May be treated with RARC, provided oncologic principles are strictly respected.
	Pure squamous cell carcinoma	RARC is a valid option when radical resection is indicated.
	Small cell neuroendocrine carcinoma	Indicated after NAC, maintaining strict oncologic principles.
**Palliative Indication**	Locally advanced or metastatic disease without curative potential	RARC may be considered for symptomatic control.
	Massive, intractable hematuria	May justify palliative RARC.
	Refractory pelvic pain, urinary fistulas, or symptom control	Goal: symptom relief and improvement of quality of life.

MIBC – Muscle-invasive bladder cancer; RARC – Robot-assisted radical cystectomy; ORC – Open radical cystectomy; TURBT – Transurethral resection of bladder tumor; NAC – Neoadjuvant chemotherapy

According to our consensus, contraindications to RARC include extensive tumor invasion of the pelvic wall, peritoneal carcinomatosis, severe ventilatory impairment incompatible with pneumoperitoneum, and technical inability to achieve safe patient positioning. Relative contraindications—dependent on surgical experience and institutional expertise—include prior pelvic surgery or radiotherapy, very high tumor burden, bulky fixed lymphadenopathy, and selected non-urothelial histology associated with unfavorable prognosis, all of which require careful individualized assessment (1, 2).

The panelists also emphasized that significant medical comorbidities—such as chronic obstructive pulmonary disease, heart failure, morbid obesity (BMI > 35 kg/m²), and extensive prior abdominal surgery with severe adhesions—may complicate robotic access, require advanced surgical expertise, or increase the risk of perioperative complications. These factors should be carefully weighed during surgical planning (1, 2).

Our consensus aligns with the available evidence, which suggests that patients with greater clinical frailty or an anesthetic risk classification of ASA ≥ III may achieve perioperative and oncologic outcomes comparable to those of lower-risk patients when managed with the robotic approach in high-volume centers. (7).

In addition, structured assessment of patient frailty is recommended as part of the preoperative evaluation. Frailty has emerged as an independent predictor of postoperative complications, prolonged hospitalization, and mortality in patients undergoing radical cystectomy. Our panelists agree that comprehensive geriatric assessment tools—including evaluation of functional status, cognition, nutritional status, and comorbidity burden—may provide more accurate risk stratification than chronological age alone. Importantly, frailty should not be considered an absolute contraindication to RARC. On the contrary, there was clear consensus that elderly and/or frail patients may benefit from a minimally invasive approach when managed in experienced, high-volume centers.(1, 2).

Obesity, particularly morbid obesity, represents a relevant clinical scenario in which RARC may offer potential perioperative advantages, provided that the procedure is performed in experienced, high-volume centers (8-10).

#### ERAS Protocols and Perioperative Management

The systematic implementation of Enhanced Recovery After Surgery (ERAS) protocols is associated with reduced surgical stress, faster functional recovery, shorter length of hospital stay, and a lower incidence of postoperative complications. Nearly all panelists routinely adopt ERAS principles in their clinical practice (Table-2).

**Table 2:** Perioperative recommendations in RARC.

Domain	Consensus Recommendation	Comment / Rationale
**ERAS Protocols**	Systematic implementation of ERAS protocols in RARC is recommended.	Associated with reduced surgical stress, faster functional recovery, shorter hospital stay, and lower complication rates.
**Functional Stratification**	Structured functional assessment and comprehensive geriatric evaluation are recommended in elderly or frail patients.	Particularly in patients ≥70 years or ASA III–IV.
	Elderly/frail patients may benefit from the robotic approach.	Potentially less surgical trauma and improved recovery.
**Nutritional Assessment**	Multifactorial preoperative nutritional assessment is recommended.	Up to one-third of patients present nutritional risk, associated with higher morbidity and mortality.
	Assessment should include BMI, clinical, and biochemical parameters.	Early identification of malnutrition.
	Preoperative immunonutrition for 5–7 days before surgery is recommended.	Arginine, glutamine, omega-3, and nucleotides may reduce infectious complications.
**Bowel Preparation**	Routine mechanical bowel preparation is not recommended.	A low-residue liquid diet may be used the day before surgery.
**Perioperative Fasting**	Solids allowed until 6 hours before; clear carbohydrate-rich fluids until 2 hours before anesthesia.	Aligned with ERAS guidelines.
	Early oral feeding (within the first 24 hours) is recommended.	Associated with reduced postoperative ileus.
	Postoperative chewing gum may be used.	Stimulates peristalsis.
**VTE Prophylaxis**	Intraoperative pneumatic compression and postoperative compression stockings are recommended.	Reduces thromboembolic risk.
	Pharmacologic thromboprophylaxis is recommended for up to 4 weeks.	Initiation between 6–24 hours after surgery.
	LMW heparin is preferred; apixaban (prophylactic dose) may be an alternative.	Institutional variation applies.
**Anesthetic Management**	Combined anesthesia (general + regional) is recommended.	Contributes to reduced ileus and improved recovery.
	Restrictive, goal-directed fluid therapy is recommended.	Reduces bowel edema.
	Opioid-sparing or opioid-free protocols are recommended.	Associated with fewer pulmonary complications and gastrointestinal dysfunction.
**Antimicrobial Prophylaxis**	Antibiotic prophylaxis is recommended for up to 24 hours after incision.	Prolonged prophylaxis shows no additional benefit.
	Continuation of antibiotics until drain/catheter removal is not recommended.	Increase risk of resistance and adverse events.
	Common regimens: 2nd/3rd generation cephalosporin + metronidazole; or ampicillin-sulbactam; or piperacillin-tazobactam.	Adjust according to institutional profile.
	Colonization with multidrug-resistant organisms requires tailored adjustment based on prior cultures.	Individualized management.
**Surgical Drainage**	Routine placement of pelvic surgical drains is commonly performed.	The benefit is still under debate
**Urinary Drainage**	No consensus on the ideal urinary drainage model.	Varies according to type of diversion.
	In ONB, adequate temporary drainage is recommended.	Protects urinary anastomosis.

BMI – Body Mass Index; LMW – Low-molecular-weight; ONB – Orthotopic Neobladder

#### Nutritional Aspects

It is estimated that up to one-third of patients undergoing this procedure present with nutritional risk, a condition associated with increased postoperative complications, impaired functional recovery, and higher mortality rates (11). An overwhelming majority of panelists recommend that nutritional assessment should be comprehensive and multifactorial, incorporating body mass index (BMI) as well as relevant clinical and biochemical parameters (11). Immunonutrition—including arginine, glutamine, omega-3 fatty acids, and nucleotides—has been associated with a reduction in infectious complications and improved metabolic response to surgical trauma. The panel recommends preoperative nutritional supplementation with immunonutrients for a minimum period of 5 to 7 days prior to radical cystectomy (12, 13).

Sarcopenia is a frequent finding in patients with bladder cancer undergoing radical cystectomy, with an estimated prevalence of half of patients, as demonstrated by a recent meta-analysis. The presence of sarcopenia was independently associated with worse oncological outcomes, with a significant increase in the risk of all-cause mortality, consolidating its role as a relevant prognostic marker in this population. Systematic assessment of body composition — preferably through computed tomography — along with nutritional evaluation in the preoperative setting of patients candidates for radical cystectomy, may identify individuals at higher risk (14).

#### Bowel Preparation and Perioperative Fasting in Robot-Assisted Radical Cystectomy

Routine mechanical bowel preparation is no longer recommended. As an alternative, a low-residue liquid diet may be adopted on the day prior to surgery.

Regarding preoperative fasting, consensus supports discontinuation of solid food intake up to 6 to 8 hours before the procedure and clear liquids up to 2 hours before anesthetic induction, including carbohydrate-containing beverages. This approach departs from the traditional concept of prolonged absolute fasting (15). Early postoperative oral feeding within the first 24 hours is also recommended (15). Chewing gum may be used as an adjunct to stimulate bowel motility and reduce the risk of postoperative ileus.

#### Venous Thromboembolism Prophylaxis

All panelists recommend the use of intermittent pneumatic compression devices from the beginning of surgery, followed by postoperative graduated compression stockings, combined with extended pharmacologic thromboprophylaxis for up to four weeks after RARC (15, 16). Pharmacologic thromboprophylaxis should be initiated between 6 and 24 hours after surgery. In the consensus, most specialists favored the use of low-molecular-weight heparin; however, some reported the use of oral anticoagulants, such as apixaban, at a prophylactic dose of 2.5 mg twice daily (17, 18). The review provides evidence that a fully intracorporeal approach during RARC may be associated with a lower thrombotic and inflammatory risk, potentially due to a reduced systemic inflammatory response, decreased postoperative pain, and faster functional recovery (19). Thromboprophylaxis is particularly important in patients who have received neoadjuvant chemotherapy, as venous thromboembolism (VTE) rates are higher in this population compared with those treated with cystectomy alone (12% vs. 8%) (18, 20). Almost all panelists recommend early postoperative physiotherapy, including both respiratory and mobilization exercises, during the in-hospital period.

#### Anesthetic Management, Fluid Therapy, and Opioid Use

The consensus recognizes that the adoption of combined anesthesia (general anesthesia plus regional techniques) and goal-directed, restrictive fluid management contributes to reduced intestinal edema, lower risk of postoperative ileus, and improved gastrointestinal recovery.

Opioid-sparing or opioid-free anesthetic protocols are recommended within ERAS pathways, as they are associated with a lower incidence of pulmonary complications, reduced postoperative ileus, and improved gastrointestinal recovery, potentially contributing to shorter hospital stay. In patients requiring perioperative opioid administration, the use of alvimopan may be considered, as it has been shown to accelerate bowel function recovery and reduce length of hospitalization (15).

#### Antimicrobial Management

Antibiotic selection should be guided by the institutional microbiological profile, prior colonization status, and individual patient risk factors, including previous hospitalizations, recent antibiotic exposure, and the type of urinary diversion performed. In addition, there was clear consensus among all panelists that RARC is associated with lower rates of surgical site infections and shorter length of hospital stay compared with the open approach.

A recent randomized clinical trial demonstrated that 24-hour antibiotic prophylaxis was non-inferior to prolonged prophylaxis (continued until removal of catheters and stents) in preventing surgical site infection within 90 days after cystectomy with urinary diversion (21). Among panelists, there was no consensus regarding the duration of antibiotic prophylaxis, which varied from up to 24 hours to 5 days, until hospital discharge, or until removal of catheters. However, current evidence supports limiting antibiotic prophylaxis to no more than 24 hours after surgical incision. Routine continuation of antibiotics until removal of drains, urinary catheters, or ureteral stents is not recommended by the majority of panelists. Prolonged use is associated with increased risk of bacterial resistance, drug-related adverse events, Clostridioides difficile infection, and higher hospital costs (1, 15, 21).

Recommended regimens include a second- or third-generation cephalosporin plus metronidazole, ampicillin–sulbactam, or piperacillin–tazobactam in higher-risk settings. Antibiotic prophylaxis should be administered 30–60 minutes before skin incision, with intraoperative redosing based on the drug's half-life and procedure duration. Prolonged antibiotic use beyond 24 hours is not recommended.

The panel agrees that, in cases of orthotopic neobladder reconstruction, an additional prophylactic antibiotic dose at the time of urethral catheter removal may be considered, according to institutional protocols. In patients with documented colonization by multidrug-resistant organisms, antibiotic selection should be tailored based on prior culture results. Asymptomatic patients with a positive preoperative urine culture should receive targeted antimicrobial therapy directed at the identified microorganism prior to RARC (1, 21).

#### Surgical and Urinary Drainage

Routine placement of pelvic surgical drains was reported by most panelists. However, no consensus was reached regarding the optimal type of drain, reflecting institutional variability and the influence of the type of urinary reconstruction performed. Routine use of nasogastric tubes was also discouraged.

With respect to urinary drainage, although the optimal duration of ureteral stents or catheters remains a matter of debate, no consensus was reached among panelists regarding the routine use, type, or duration of urinary drainage in either ileal conduit or orthotopic neobladder reconstruction. Similarly, no consensus was achieved regarding several technical aspects of ureteroenteric anastomosis, including the choice between Bricker and Wallace techniques, the extent of ureteral spatulation, continuous versus interrupted suturing, the routine use of transanastomotic ureteral stents, and the optimal duration of stenting. These findings reflect the considerable variability in surgical practice and the limited high-level evidence supporting one specific approach over another. Preservation of ureteral vascularization appears to be more important than the choice of ureteroenteric anastomotic technique (Bricker versus Wallace) or stenting strategy in minimizing the risk of ureteroenteric strictures and related complications. (15, 19, 22). Aditionally, no consensus was achieved regarding the optimal duration of urethral catheterization following orthotopic neobladder reconstruction.

## ROBOTIC VS. OPEN APPROACH IN ONCOLOGIC OUTCOMES

Randomized clinical trials and contemporary meta-analyses confirm comparable outcomes between robotic and open radical cystectomy in terms of overall survival (OS), recurrence-free survival (RFS), locoregional disease control, and rates of positive surgical margins (4, 19, 23).

### Surgical Margins

Robot-assisted radical cystectomy (RARC) demonstrates positive surgical margin (PSM) rates comparable to those of open radical cystectomy. These rates vary according to disease stage, ranging from approximately 3% to 8% in organ-confined disease and from 15% to 20% in locally advanced cases (1, 2, 4, 19).

The main independent predictors of positive surgical margins include advanced clinical and pathological stage, lymph node positivity, variant histology (such as micropapillary or sarcomatoid features), lymphovascular invasion, lack of response to neoadjuvant chemotherapy, and a history of prior pelvic radiotherapy (1, 2, 4, 19, 23).

When a positive ureteral margin is identified on intraoperative frozen section analysis (FSA), sequential cranial ureteral resections are recommended until carcinoma in situ or invasive disease is excluded. (24). Despite evidence that routine intraoperative FSA of the urethral margin reduces the risk of positive surgical margins and, when combined with the absence of prostatic stromal invasion, enables safe urethral preservation in candidates for orthotopic neobladder reconstruction, only 48% of panelists reported performing it routinely, while others reserve its use for cases of locally advanced disease (1, 2).

### Extent of Pelvic Lymphadenectomy

Pelvic lymph node dissection (PLND) remains a fundamental component of radical cystectomy, providing both staging and potential therapeutic benefit. The extent of lymphadenectomy continues to be a matter of debate, particularly in the setting of robot-assisted radical cystectomy (RARC) (4, 19).

The panel supports the performance of at least an extended pelvic lymph node dissection in patients undergoing RARC, given its established role in accurate staging and its potential impact on oncologic outcomes. Although randomized evidence has not demonstrated a significant overall survival benefit with super-extended templates compared to extended dissection, improved staging accuracy remains a consistent finding. The anatomical boundaries are defined proximally by the bifurcation of the common iliac artery, distally by the node of Cloquet (Cooper's ligament), laterally by the genitofemoral nerve, medially by the pelvic visceral structures, and posteriorly by the obturator fossa. The dissection includes the external iliac, internal iliac, and obturator lymph node basins (1, 2, 25).

Extended lymphadenectomy maintains the same distal and lateral boundaries but expands proximally to include the entire length of the common iliac arteries up to the aortic bifurcation, as well as the presacral region. Super-extended lymphadenectomy extends beyond the common iliac bifurcation and includes the lower para-aortic and para-caval lymph nodes up to the level of the inferior mesenteric artery (26).

Recent evidence suggests that performing PLND before or after radical cystectomy results in comparable lymph node yield, blood loss, and operative outcomes. In robotic surgery, however, PLND performed before cystectomy may facilitate the procedure by reducing cystectomy time, overall operative time, and blood loss, supporting the use of either sequence according to surgeon preference or technical considerations (27).

The panel emphasizes that the quality and completeness of lymphadenectomy are critical factors, potentially more relevant than the absolute anatomical extent. Standardization of surgical templates, meticulous dissection, and adequate lymph node yield are essential to optimize oncologic outcomes. In addition, the panel considers robot-assisted lymphadenectomy to be a safe and feasible approach, even in the presence of bulky nodal disease or following perioperative systemic therapy, provided that the procedure is performed in experienced, high-volume centers.

### Perioperative Systemic Therapy in the Management of Muscle-Invasive Bladder Cancer (MIBC)

The vast majority of panelists considered RARC to be safe following perioperative systemic therapy. In addition, the panel supports the routine use of perioperative systemic therapy in eligible patients. This position is consistent with recommendations from the European Association of Urology (EAU) and other major international societies, which endorse perioperative systemic therapy as a standard component of care in appropriately selected patients (1), as well as the American Urological Association (AUA/ASCO/SUO) (2) and National Comprehensive Cancer Network (NCCN) (28), recommend the use of perioperative chemotherapy and/or immunotherapy for all eligible patients with cT2–T4a N0 M0 tumors. Adjuvant chemotherapy (AC) is reserved for patients with high-risk pathological findings after surgery, such as pT3/pT4 disease or lymph node involvement, particularly in the absence of prior chemotherapy (Table-3).

**Table 3:** Perioperative Systemic Treatment (MIBC).

Domain	Consensus Recommendation	Clinical Comment
**Neoadjuvant Chemotherapy (NAC)**	Recommended for cT2–T4a N0 M0 eligible patients.	Supported by EAU, AUA/ASCO/SUO, and NCCN guidelines.
	Cisplatin-based regimens (GC or MVAC).	Dose-dense MVAC improves PFS
**Adjuvant Chemotherapy (AC)**	Indicated in pT3/pT4 or N+ patients without prior NAC.	Based on pathological risk stratification.
**Adjuvant Nivolumab**	Recommended in high-risk patients (pT2–4 and/or N+).	Improves DFS and OS
**Durvalumab + GC**	Promising perioperative strategy.	Potential paradigm shift in management.
**NAC and Surgical Complications**	NAC does not increase surgical morbidity.	Multicenter evidence (30- and 90-day outcomes).
**Surgical Timing**	Surgery within 10–12 weeks after NAC is considered safe.	Does not compromise oncologic or surgical outcomes.
**Enfortumab Vedotin + Pembrolizumab**	Improves EFS, OS, and pCR rates.	Strongly recommended by the 2026 EAU Guidelines as perioperative therapy for patients with MIBC who are ineligible for cisplatin-based chemotherapy

**EAU** – European Association of Urology; **AUA** – American Urological Association; **ASCO** – American Society of Clinical Oncology; **SUO** – Society of Urologic Oncology; **NCCN** – National Comprehensive Cancer Network; **GC** – Gemcitabine plus Cisplatin; **MVAC** – Methotrexate, Vinblastine, Doxorubicin, and Cisplatin; **N+** – Lymph Node-Positive Disease; **NAC** – Neoadjuvant Chemotherapy; **DFS** – Disease-Free Survival; **OS** – Overall Survival; **EFS** – Event-Free Survival; **pCR** – Pathological Complete Response

Cisplatin-based regimens remain the standard for neoadjuvant chemotherapy (NAC), with gemcitabine plus cisplatin (GC) and MVAC (methotrexate, vinblastine, doxorubicin, and cisplatin) being the most extensively studied protocols (1, 2, 28). The dose-dense MVAC regimen has demonstrated improved progression-free survival (29).

Adjuvant nivolumab is approved and has been shown to significantly improve disease-free survival and overall survival in high-risk patients (pT2–4 and/or N+) following cystectomy (1, 30). Adjuvant nivolumab should be offered to selected patients who did not receive or declined platinum-based chemotherapy.

The use of durvalumab in combination with gemcitabine plus cisplatin prior to surgery, followed by adjuvant maintenance therapy, has been shown to improve overall survival and event-free survival compared with chemotherapy alone and has been adopted in several countries in Latin America (31).

Retrospective studies and meta-analyses have consistently demonstrated that neoadjuvant chemotherapy is not associated with an increased risk of surgical complications. A multicenter study further evaluated this association (32) In a cohort of 878 patients (302 of whom received NAC), no significant differences were observed in 30- and 90-day complication rates between groups. An interval of 10 to 12 weeks between completion of chemotherapy and surgery does not compromise surgical or oncologic outcomes (33). Most panelists consider that neoadjuvant chemotherapy does not increase surgical morbidity and can be regarded as safe following neoadjuvant chemoimmunotherapy.

The perioperative combination of enfortumab vedotin plus pembrolizumab has demonstrated significant improvements in event-free survival, overall survival, and pathological complete response rates in patients with MIBC who are ineligible for cisplatin-based chemotherapy (34). Based on these findings, this regimen has been incorporated into the 2026 EAU Guidelines as a strongly recommended perioperative treatment option for cisplatin-ineligible patients. The panel acknowledges the relevance of these findings and their potential to reshape perioperative treatment strategies in MIBC.

The majority of panelists agreed that robot-assisted radical cystectomy should be performed within 30 to 60 days following completion of neoadjuvant systemic therapy, provided that the patient's clinical and laboratory conditions are adequate.

## FUNCTIONAL ASPECTS

### Nerve-Sparing Approach in Men

RARC provides enhanced anatomical visualization compared with open surgery and facilitates preservation of the neurovascular bundles as well as the endopelvic fascia (Endopelvic Fascia Sparing – EPFS). This approach has been associated with improved urinary continence and sexual function outcomes (35, 36). The majority of panelists agreed that preservation of the endopelvic fascia, puboprostatic ligaments, dorsal venous complex, and neurovascular bundles, using an athermal dissection technique, is associated with improved urinary continence and erectile function outcomes (Table-4) (35).

**Table 4:** Urinary Diversion Strategy in RARC.

Technical Domain	Consensus Recommendation	Technical Considerations
**Ileal Conduit**	Remains the most reproducible and reliable diversion.	Preferred in frail patients, significant comorbidities, or limited functional reserve.
**Neobladder**	Recommended in selected patients with favorable oncologic and functional criteria. Should be strict and individualized.	Requires preserved urethra, negative margins, adequate sphincter function, and renal function (CrCl > 50–60 mL/min). Motivated patients with ability to perform self-care and possible intermittent catheterization.
**Contraindications to Neobladder**	Renal impairment, bowel disease, sphincter dysfunction, and inability to perform self-catheterization should be considered relative contraindications (higher risk of metabolic complications and suboptimal functional outcomes).	
**Intracorporeal Diversion (ICUD)**	Increasingly adopted in high-volume centers.	Associated with less bowel manipulation and improved perioperative recovery.
**Extracorporeal Diversion (ECUD)**	Remains a valid alternative.	May be preferred in selected complex or high-risk cases.
**Mesentery-Sparing Technique**	Recommended during intracorporeal reconstruction.	Preserves vascular supply, reduces mesenteric defects, and lowers risk of internal hernias.
**Bilateral Cutaneous Ureterostomy**	Valid alternative in selected patients.	Consider frail patients, high surgical risk, limited life expectancy, or solitary kidney.

The best candidates are younger patients with organ-confined disease (≤pT2) and no involvement of the bladder neck or urethra. In cases of locally advanced, multifocal, or high oncologic risk disease, tumor control remains the priority, and functional preservation strategies should be avoided (1, 35-37). The vast majority of panelists reported routinely using polymer clips for ligation of the lateral pedicles, whereas only a minority prefer energy-based devices for this purpose.

Sexual dysfunction is a common treatment-related consequence of urological cancers and should be addressed during pre-treatment counseling (38).

### Urethra / Urethrectomy

The panel recommends circumferential urethral dissection should be performed at the level of the prostatic apex using blunt dissection and only after complete bladder mobilization. Bladder decompression is recommended, followed by placement of a polymer clip or ligature as proximally as possible on the urethra to prevent urinary spillage.

In candidates for orthotopic neobladder reconstruction, preservation of the maximal urethral length is recommended whenever oncologically safe (6, 39). The urethra-ileal anastomosis should be performed using a technique similar to the Van Velthoven method (40) and, in most cases, should be completed prior to isolation of the neobladder segment.

The AUA/ASCO/SUO guidelines further address this aspect (2) and also the EAU Guidelines 2025 (1) Current evidence and international guidelines recognize that prophylactic urethrectomy should not be routinely performed. According to the available literature and the present consensus, urethrectomy is primarily indicated in patients with confirmed urethral involvement, advanced tumor stage (T3–T4), presence of carcinoma in situ (CIS), positive intraoperative urethral frozen section, or documented residual urethral disease. There was strong consensus among panelists that concomitant urethrectomy should be reserved for cases with confirmed involvement or high-risk features for urethral disease, although some differences in decision-making were observed between male and female patients (1, 2, 41).

The decision to perform intraoperative frozen section analysis of the urethral margin should take into account the individual oncologic risk and the experience of the surgical team. When a positive urethral margin is identified, additional resection may convert the margin to negative, which has been associated with a lower risk of urethral recurrence. Conversely, a final positive urethral margin is associated with a significantly increased risk of urethral recurrence within the first three years of follow-up (1, 42).

Regarding the panelists’ responses, in men, the highest levels of agreement were observed for documented urethral invasion and distal urethral involvement, while the presence of CIS was also frequently considered. Other factors, such as bladder neck involvement and multifocal disease, were less consistently regarded as absolute indications, suggesting a more individualized approach in these scenarios. In contrast, among women, a more consistent pattern of responses was observed, with confirmed urethral involvement, urethral compromise, and the presence of CIS reaching higher levels of agreement among panelists. Trigonal involvement was also more frequently considered relevant, whereas multifocal disease appeared to have a more limited impact on decision-making.

The procedure may be performed through an abdominal or perineal approach, particularly in cases of extensive local invasion. The guidelines emphasize that the decision to perform urethrectomy should be selective and based on individual oncologic risk, intraoperative assessment of surgical margins, and the planned urinary diversion strategy. (1, 2, 43). In addition, assessment of vaginal support and the impact of urethral resection on pelvic stability should be considered, since the concomitant removal of the urethra and anterior vaginal wall may significantly alter pelvic anatomy and increase the risk of functional and structural complications."

### RARC in Women and Concomitant Hysterectomy and Vaginal Wall Resection

In postmenopausal women with an atrophic uterus or endometrial abnormalities, concomitant hysterectomy remains the standard approach. Conversely, in younger patients without cervical or anterior vaginal wall invasion, uterine preservation may be considered, provided that oncologic principles are strictly maintained. In this context, there was consensus among panelists that younger women, particularly those aiming to preserve sexual function, may benefit from RARC when organ-preserving strategies are appropriately applied (Table-4).

Resection of the anterior vaginal wall and urethra during female radical cystectomy is primarily indicated in cases of locally advanced disease or when there is evidence of tumor invasion compromising oncologic safety and the achievement of negative surgical margins, particularly in T3 or T4 tumors with extension to adjacent pelvic structures and/or aggressive histologic variants (1, 2, 43).

Preservation of the anterior vaginal wall and urethra is associated with orthotopic neobladder reconstruction. Maintenance of the urethra, vaginal support structures, and surrounding fascia reduces the risk of vaginal dehiscence, pelvic organ prolapse, and vaginal failure following cystectomy (43, 44). Nerve-sparing in selected women is appropriate for patients undergoing intracorporeal orthotopic neobladder reconstruction, with the aim of optimizing functional outcomes, particularly urinary continence. The consensus panel recognizes that, when oncologically safe, female nerve-sparing and organ-preserving strategies may also contribute to better sexual and pelvic functional outcomes. Preservation of the autonomic pelvic plexus, anterior vaginal wall, uterus and supporting structures has been associated in the literature with reduced vaginal shortening and dyspareunia, improved vaginal lubrication, better preservation of sexual function, and lower risk of pelvic floor dysfunction after radical cystectomy (44).

### Vaginal Closure and Prevention of Prolapse and Fistula

There was consensus among panelists that uterine preservation and maintenance of its supporting structures may improve sexual function, preserve pelvic floor support, and reduce the risk of prolapse following orthotopic neobladder reconstruction. Patient preference should be explicitly considered in the decision-making process.

Panelists agreed that preservation of the anterior vaginal wall facilitates orthotopic neobladder reconstruction. In addition, this approach was considered to be associated with improved body image, psychosexual outcomes, sexual function, patient-reported outcomes, and overall quality of life. Furthermore, proper vaginal closure techniques using absorbable sutures, along with reinforcement of the anterior compartment, are considered important to reduce late complications and optimize functional outcomes after continent urinary reconstruction (44, 45). In selected cases, peritoneal coverage or the use of pedicled omental flaps over the suture line may be considered to reduce the risk of neobladder–vaginal fistula. Techniques such as prophylactic sacrocolpopexy or suspension to the pectineal (Cooper's) ligament, combined with primary vaginal closure, represent effective strategies to prevent vaginal vault prolapse (44, 45).

### Posterior Reconstruction Technique

The aim of this technique is to restore pelvic floor anatomy, minimize tension on the anastomosis, and provide support to the neobladder. The consensus panel recognized that posterior reconstruction (Rocco's stitch) offers technical benefits, particularly in reducing tension on the urethra-ileal anastomosis and contributing to its stabilization. However, no consensus was reached regarding its impact on early functional recovery or long-term urinary continence outcomes (40).

## URINARY RECONSTRUCTION

The choice of urinary diversion following radical cystectomy should be individualized and based on shared decision-making, taking into account oncologic, anatomical, and functional factors, as well as the patient's overall condition, cognitive status, and quality-of-life expectations.

The panel agrees that the ileal conduit remains the most reproducible and technically reliable form of urinary diversion, particularly in patients with significant comorbidities, frailty, limited functional or cognitive reserve, or higher surgical risk. Although advanced age alone is not an absolute contraindication to orthotopic neobladder, it is associated with an increased risk of complications and lower likelihood of optimal functional outcomes and should therefore be considered in the decision-making process.

Orthotopic neobladder is preferably indicated in patients with a preserved urethra and negative margins, adequate sphincter function, and sufficient renal function—typically defined as a creatinine clearance above 50–60 mL/min—given the metabolic risks associated with intestinal urinary reconstruction. Motivated patients with good functional status and the ability to understand and perform clean intermittent catheterization, if necessary, are more likely to achieve favorable functional outcomes.

In women, preservation of the urethra, anterior vaginal wall, and adequate urethral support are key determinants of eligibility and functional success. The panel emphasizes that careful patient selection is critical to optimize both oncologic safety and functional outcomes.

Major contraindications to continent urinary diversion include significant renal impairment, relevant intestinal disease, sphincter dysfunction, inability to perform self-catheterization, neurological disorders affecting voiding function, and, in selected cases, prior pelvic radiotherapy. In addition, clinically significant hepatic dysfunction—particularly conditions associated with impaired ammonia metabolism—should be considered a contraindication, given the increased risk of hyperammonemia and metabolic complications related to urinary contact with intestinal segments. These factors are associated with increased risks of metabolic disturbances, urinary retention, and functional failure, and should be carefully considered during patient selection.

Various neobladder techniques have been described in the literature, without definitive evidence demonstrating clear superiority of one approach over another in terms of oncologic or functional outcomes. As such, the choice of technique remains largely dependent on surgeon experience and institutional expertise.

The panel recommends preoperative stoma site marking in patients undergoing ileal conduit (Bricker) urinary diversion. Marking should be performed with the patient awake, ideally in both sitting and standing positions, to optimize placement and reduce postoperative complications. This process may be performed by a trained stoma therapist or by the surgeon.

The panel also recognizes bilateral cutaneous ureterostomy as a valid alternative in selected scenarios, particularly in frail patients, those with high anesthetic risk, limited performance status, reduced life expectancy, or a solitary functioning kidney (46, 47).

### Intracorporeal versus Extracorporeal Reconstruction

From a perioperative perspective, intracorporeal urinary diversion (ICUD) has been associated with a lower incidence of gastrointestinal complications, reduced rates of hospital readmission at 30 and 90 days, and a decreased risk of surgical site infection (48). Additionally, ICUD may facilitate faster gastrointestinal recovery, including earlier return of bowel function, earlier resumption of oral intake, and a reduction of approximately one to two days in length of hospital stay when compared with extracorporeal urinary diversion (ECUD) (49, 50); however, no significant differences have been consistently demonstrated between ICUD and ECUD in terms of functional outcomes, including daytime or nighttime continence, or recovery of erectile function (49, 50).

The panel acknowledges that the adoption of ICUD is increasing, particularly in high-volume centers, and reflects a trend toward complete minimally invasive management of radical cystectomy.

Importantly, fully intracorporeal urinary diversion avoids extensive bowel externalization and manipulation. In this context, the panel reached consensus regarding the adoption of mesentery-sparing techniques, which aim to preserve mesenteric vascular integrity, minimize the creation of mesenteric defects, and reduce the risk of internal hernias (51).

### Neobladder Techniques Utilized by the Panelists

All panelists reported using the terminal ileum for orthotopic neobladder reconstruction. The terminal ileum remains the preferred segment for both ileal conduit and neobladder construction due to its favorable distensibility, lower peristaltic activity, and suitability for intracorporeal manipulation with robotic and laparoscopic instruments. However, considerable heterogeneity was observed regarding the specific techniques employed in clinical practice. The most frequently reported techniques were the modified Studer neobladder (52) and the "Bordeaux" variant (53), each utilized by approximately 40% of respondents. Other techniques were significantly less commonly used. Notably, 13.8% of panelists reported not performing intracorporeal orthotopic neobladder reconstruction in their practice. These findings highlight the lack of universal technical standardization and reinforce that the choice of technique remains largely dependent on surgeon experience and institutional expertise.

### Ileal Segment Length for Intracorporeal Neobladder

Regarding the length of ileum used for intracorporeal orthotopic neobladder construction, variability among specialists was also observed. Most panelists reported using segments ranging from 45 to 60 cm, with 45 cm being the most frequently selected option (34.5%), followed by 60 cm (27.6%) and 50 cm (24.1%). Longer segments, such as 55 cm or greater than 70 cm, were less commonly utilized, while 40 cm was rarely selected. These findings suggest a practical trend concentrated between 45 and 60 cm, although without formal consensus, reflecting adaptation of the technique to individual surgeon preference as well as patient-specific anatomical and functional characteristics.

### Urinary Continence and Voiding Function After Robotic Neobladder

Available data demonstrate that urinary continence rates following orthotopic neobladder reconstruction in robotic radical cystectomy are overall comparable to those observed with the open approach, although considerable heterogeneity exists among studies due to differences in study design, continence definitions, follow-up duration, and surgical techniques. In general, when stricter definitions and longer follow-up periods are considered, daytime continence rates range approximately from 60% to 90%, while nighttime continence ranges from 40% to 70%.

These outcomes are multifactorial and depend on factors such as nerve preservation, residual urethral length, sphincter function integrity, reservoir capacity and low-pressure characteristics, as well as the ability to achieve complete bladder emptying. Temporal evolution also plays a significant role, with progressive improvement in functional outcomes observed over the first 12 to 24 months following surgery (54). Retrospective studies with limited sample sizes evaluating nerve-sparing RARC report mean daytime continence rates of approximately 90% and nighttime continence rates around 80% (35, 37, 54).

In a systematic analysis involving different orthotopic neobladder techniques performed intracorporeally in high-volume centers, approximately 88% of patients achieve daytime continence at 1 year (defined as the use of no more than one pad per day), with variability across techniques—for example, 88% for Studer neobladder, 90% for Y-shaped neobladder, and up to 95% for Pyramid and Hautmann techniques. Night-time continence demonstrates greater variability, with generally lower rates that are technique-dependent, being highest in the Hautmann neobladder. The need for clean intermittent catheterization is relatively low, ranging from 0% to 7% in most series, reflecting adequate voiding function in the majority of patients (55).

Overall, the best continence outcomes are observed in high-volume centers and in series with greater technical standardization, and are influenced by factors such as reconstruction technique, surgical experience, and structured functional rehabilitation protocols. The panel recommends that patients be counseled preoperatively regarding the need for pelvic floor rehabilitation and the potential requirement for clean intermittent catheterization during the early phases of functional recovery.

Intracorporeal urinary diversion requires extensive intestinal mobilization, a step that can be particularly challenging when associated with the mesentery-sparing technique (51). This approach aims to preserve mesenteric vascularization, limiting excessive bowel deflection and reducing the risk of internal hernias. In addition, preserving segmental intestinal perfusion may decrease the likelihood of anastomotic complications, even though it limits mesenteric mobilization and contributes to greater reconstructive safety.

## COMPLEX CASES IN ROBOT-ASSISTED RADICAL CYSTECTOMY

### Obesity

Obesity represents a relevant technical challenge during robot-assisted radical cystectomy (RARC), particularly due to limited visualization, reduced bowel mobility, and restricted instrument maneuverability related to increased abdominal wall thickness and deeper pelvic anatomy. In this context, the panel recommends technical adjustments to optimize surgical exposure and stability, including more cranial positioning of robotic trocars, controlled increases in pneumoperitoneum pressure. Intracorporeal urinary diversion may further enhance visualization and facilitate surgical control in these patients.

Despite the increased technical complexity and higher baseline risk of perioperative complications, there was agreement among panelists that patients with elevated BMI, including those with BMI >40 kg/m², may benefit from RARC, likely due to reduced abdominal wall trauma, lower wound-related morbidity, decreased postoperative pain, and faster functional recovery. In appropriately selected cases, the robotic approach may also facilitate orthotopic neobladder reconstruction, preserving the option of continent urinary diversion despite the challenges traditionally associated with open surgery in obese individuals. Accordingly, obesity should not be considered an absolute contraindication to RARC but rather a condition that requires meticulous preoperative planning and management by experienced surgical teams in high-volume centers (8-10).

### Locally Advanced and High-Volume Tumors

In patients with locally advanced bladder cancer (T3–T4) considered resectable, the panel recognizes that RARC may be a feasible approach in high-volume centers with experienced surgeons, even in the presence of adjacent organ involvement or when extended resections are anticipated. Adequate preoperative imaging is essential to guide surgical planning, including anticipation of extended resections and early definition of the urinary diversion strategy. In anterior or lateral tumors, initiating dissection on the contralateral side may improve vascular control and anatomical orientation. Direct tumor traction and excessive manipulation of bulky bladder masses should be avoided.

In cases of significant bleeding, loss of oncologic planes, or unexpected extensive invasion, conversion to open surgery should be promptly considered to ensure patient safety and oncologic adequacy. In the presence of pelvic sidewall invasion, the panel noted considerable divergence in opinion. This scenario should be regarded as a relative, experience-dependent contraindication, and many experts consider the open approach to remain an appropriate alternative (1, 2, 19, 56).

### Bulky Lymphadenopathy

Bulky pelvic lymphadenopathy can significantly distort anatomy, increase adherence to vascular structures, and raise the risk of intraoperative vascular injury.

The panel emphasizes the importance of performing clear vascular dissection above and below the enlarged lymph nodes to ensure precise identification of anatomical landmarks prior to tumor mobilization, thereby reducing the risk of complications (25, 26).

### Salvage Robot-Assisted Radical Cystectomy

Salvage RARC following prior trimodal therapy represents an increasingly common clinical scenario, accounting for approximately 6%–18% of cystectomies in high-volume centers (57).

Available evidence demonstrates higher rates of major complications (Clavien ≥ III), ranging from 20% to 32%, and positive surgical margins in approximately 10% of cases (23). Prior radiation therapy has been identified as an independent predictor of increased postoperative complications, longer operative time, and prolonged hospital stay (58).

Almost all panelists agreed that RARC is feasible and may be indicated in salvage settings. In this context, the panel recommends a lateral-to-medial dissection approach and the use of advanced energy devices. Patients with prior pelvic irradiation should be carefully counseled regarding the increased risks of bowel complications, ureteroenteric strictures, and urinary or vaginal fistulas (59).

Additionally, intraoperative fluorescence imaging with indocyanine green (ICG) may be considered to assess ureteral and bowel perfusion, potentially reducing the risk of ischemia-related complications (60).

## COST-EFFECTIVENESS ANALYSIS OF ROBOTIC RADICAL CYSTECTOMY

Robot-assisted radical cystectomy (RARC) is associated with higher initial costs, primarily related to robotic technology and longer operative time. However, when performed in high-volume centers by experienced surgeons, and combined with intracorporeal urinary diversion (ICUD) and enhanced recovery after surgery (ERAS) protocols, RARC may achieve cost-effectiveness comparable to -or even exceeding- that of open radical cystectomy.

The panel recognizes that improved perioperative outcomes, including reduced complications, shorter hospital stay, and lower readmission rates, may offset the initial procedural costs. Therefore, appropriate patient selection, surgical expertise, and institutional efficiency are key determinants of cost-effectiveness (61-63).

## EMERGING ROBOTIC AND TELEPRESENCE PLATFORMS IN RADICAL CYSTECTOMY

New robotic platforms for radical cystectomy are currently undergoing expansion and validation (64). However, the consensus recognizes that clinical evidence specifically in RARC remains limited. Continuous evaluation of safety and efficacy, team experience, and strict adherence to oncologic principles remain more critical determinants of outcomes than the specific robotic platform employed. In parallel, the evolution of telesurgery and telepresence systems represents a potential next frontier for complex oncologic procedures, including radical cystectomy. Recent literature highlights the feasibility of ultra–long-distance robotic surgery supported by high-bandwidth, low-latency networks such as 5G infrastructure, with emphasis on communication redundancy, real-time data transmission stability, and structured governance frameworks for remote implementation. Clinical guidelines for telesurgery stress rigorous validation, defined backup protocols, and clear medico-legal accountability before broader adoption. Although most published human experiences have focused on procedures such as radical prostatectomy, the same technological principles—network reliability, dual-console integration, tele-mentoring capability, and human supervision—are directly translatable to RARC. At present, telesurgery in cystectomy remains investigational, and its safe expansion will depend on robust infrastructure, institutional oversight, and preservation of core oncologic standards (65).

Finally, despite its strengths, this consensus has limitations that should be acknowledged. First, although the recommendations were informed by a review of the current literature and the clinical experience of participating experts, many aspects of radical cystectomy management remain supported primarily by observational studies rather than high-level randomized evidence. As a result, some statements reflect expert interpretation of available data rather than definitive evidence-based conclusions. In addition, practice patterns may vary across institutions depending on surgical expertise, available technology, and patient population, which may limit the generalizability of certain recommendations. As surgical techniques and perioperative management strategies continue to evolve, particularly with the increasing adoption of minimally invasive approaches and enhanced recovery protocols, these recommendations should be interpreted within the context of ongoing developments in bladder cancer care. Future prospective multicenter studies will be essential to validate and refine these recommendations

## CONCLUSIONS

Radical cystectomy remains the optimal approach of curative treatment for patients with high-risk non–muscle-invasive and muscle-invasive bladder cancer. Despite significant advances in surgical techniques, perioperative management, and patient selection, substantial variability persists in clinical practice across institutions and regions. This consensus provides a structured framework for the contemporary management of patients undergoing radical cystectomy, integrating current evidence with expert clinical experience. By addressing key aspects such as patient selection, surgical approach, perioperative optimization, and postoperative outcomes, these recommendations aim to support standardized, high-quality care and improve oncologic and functional outcomes. Future research should focus on prospective studies evaluating surgical innovations, enhanced recovery strategies, and patient-centered outcomes to further refine the role of radical cystectomy within multidisciplinary bladder cancer care.

## Data Availability

All data generated or analysed during this study are included in this published article
